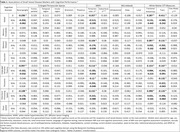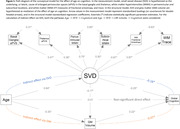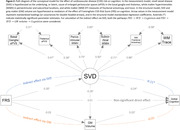# Modelling Small Vessel Disease Using Regional MRI Markers, and Mediating Effects on Cognitive Decline

**DOI:** 10.1002/alz.093701

**Published:** 2025-01-09

**Authors:** Sokratis Charisis, Tanweer Rashid, Christina S. Dintica, Mitzi M. Gonzales, Hangfan Liu, Jeffrey B. Ware, Thomas R. Austin, Paul N. Jensen, Alison E Fohner, Jordan E. Tanley, Jingzhong Ding, José A. Luchsinger, Bonnie C. Sachs, Ilya M. Nasrallah, R Nick Bryan, Kathleen M. Hayden, David A Wolk, Katya Rascovsky, Christos Davatzikos, W.T. Longstreth, Kristine Yaffe, Sudha Seshadri, Susan R. Heckbert, Timothy M. Hughes, Mohamad Habes

**Affiliations:** ^1^ Glenn Biggs Institute for Alzheimer’s & Neurodegenerative Diseases, University of Texas Health Sciences Center at San Antonio, San Antonio, TX USA; ^2^ Neuroimage Analytics Laboratory (NAL) and the Biggs Institute Neuroimaging Core (BINC), Glenn Biggs Institute for Alzheimer’s & Neurodegenerative Diseases, University of Texas Health Sciences Center, San Antonio, TX USA; ^3^ University of California, San Francisco, San Francisco, CA USA; ^4^ Glenn Biggs Institute for Alzheimer’s & Neurodegenerative Diseases, San Antonio, TX USA; ^5^ University of Pennsylvania, Philadelphia, PA USA; ^6^ University of Washington, Seattle, WA USA; ^7^ Wake Forest University School of Medicine, Winston‐Salem, NC USA; ^8^ Wake Forest University School of Medicine, Winston Salem, NC USA; ^9^ Columbia University Irving Medical Center, New York, NY USA; ^10^ Artificial Intelligence in Biomedical Imaging Laboratory (AIBIL), Center for and Data Science for Integrated Diagnostics (AI2D), Perelman School of Medicine, University of Pennsylvania, Philadelphia, PA USA; ^11^ University of Pennsylvania Health System, Philadeplphia, PA USA; ^12^ Perelman School of Medicine, University of Pennsylvania, Philadelphia, PA USA; ^13^ Artificial Intelligence in Biomedical Imaging Laboratory (AIBIL), Perelman School of Medicine, University of Pennsylvania, Philadelphia, PA USA; ^14^ University of California, San Francisco, Weill Institute for Neurosciences, San Francisco, CA USA; ^15^ Glenn Biggs Institute for Alzheimer’s & Neurodegenerative Diseases, University of Texas Health Science Center, San Antonio, TX USA; ^16^ Neuroimage Analytics Laboratory and Glenn Biggs Institute Neuroimaging Core, Glenn Biggs Institute for Neurodegenerative Diseases, University of Texas Health San Antonio, San Antonio, TX USA

## Abstract

**Background:**

The location of proposed brain MRI markers of small vessel disease (SVD) might reflect their pathogenesis and may translate into differential associations with cognition. We derived regional MRI markers of SVD and studied: (i) associations with cognitive performance, (ii) patterns most likely to reflect underlying SVD, (iii) mediating effects on the relationships of age and cardiovascular disease (CVD) risk with cognition.

**Method:**

In 891 participants from The Multi‐Ethnic Study of Atherosclerosis, we segmented enlarged perivascular spaces (ePVS), white matter hyperintensities (WMH) and microbleeds (MBs) using deep learning‐based algorithms, and calculated white matter (WM) microstructural integrity measures of fractional anisotropy (FA), trace (TR) and free water (FW) using automated DTI‐processing pipelines. Measures of global and domain‐specific cognitive performance were derived from a comprehensive cognitive evaluation based on the UDS v3 neuropsychological battery.

**Result:**

Mean (SD) age was 73.6 (7.9) years; 474 (53%) participants were women. In generalized linear models adjusted for demographics, vascular risk factors, and APOE ε4 carriership, higher basal ganglia ePVS count was associated with worse global, language, and attention cognitive performance (Table 1). Higher periventricular WMH volume was associated with worse global, delayed memory, language, phonemic, and attention performance. Higher WM FA was associated with better global, delayed memory, language, and attention performance. Higher WM TR was associated with worse global, delayed memory, language, phonemic, and attention performance. Exploratory factor analysis revealed that basal ganglia ePVS (standardized loading = 0.51), thalamus ePVS (0.43), periventricular WMH (0.85), subcortical WMH (0.65), and WM FA (‐0.73) and TR (0.84) loaded onto the same factor, likely reflecting underlying SVD. Structural equation models demonstrated that SVD mediated the effect of age on cognition (β[95%CI] = ‐0.071[‐0.088,‐0.053]) through the pathways: Age→SVD→Cognition (‐0.044[‐0.063,‐0.026]) and Age→SVD→Brain Atrophy→Cognition (‐0.006[‐0.012,‐0.002]) – Figure 1, and the effect of CVD risk on cognition (‐0.028[‐0.044,‐0.012]) through the pathways: CVD Risk→SVD→Cognition (‐0.021[‐0.031,‐0.013]) and CVD Risk→SVD→Brain Atrophy→Cognition (‐0.007[‐0.012,‐0.003]) – Figure 2.

**Conclusion:**

The location of the proposed MRI markers of SVD likely reflects distinct etiopathogenic substrates and should be considered when examining associations with cognitive or other health‐related outcomes. SVD mediates the relationships of age and CVD risk with cognition via both atrophy‐related and unrelated pathways.